# 545 A PRospective, Case-controlled Evaluation of oLIceridine for Moderate or sEVEre Pain After Burn Injury. (RELIEVE)

**DOI:** 10.1093/jbcr/irae036.179

**Published:** 2024-04-17

**Authors:** David M Hill, Yvonne Shaw, Lorraine A Todor, Aaron L Rank, Cleo C Carter, Xiangxia Liu, Mahmoud Hassouba, Sai R Velamuri

**Affiliations:** Regional One Health, Memphis, TN; University of Tennessee Health Science Center Firefighters' Burn Center at Regional One, Memphis, TN; University of Tennessee Health Science Center, Memphis, TN; Firefighters Burn Center, Regional One Health, Memphis, TN; Regional One Health, Memphis, TN; University of Tennessee Health Science Center Firefighters' Burn Center at Regional One, Memphis, TN; University of Tennessee Health Science Center, Memphis, TN; Firefighters Burn Center, Regional One Health, Memphis, TN; Regional One Health, Memphis, TN; University of Tennessee Health Science Center Firefighters' Burn Center at Regional One, Memphis, TN; University of Tennessee Health Science Center, Memphis, TN; Firefighters Burn Center, Regional One Health, Memphis, TN; Regional One Health, Memphis, TN; University of Tennessee Health Science Center Firefighters' Burn Center at Regional One, Memphis, TN; University of Tennessee Health Science Center, Memphis, TN; Firefighters Burn Center, Regional One Health, Memphis, TN; Regional One Health, Memphis, TN; University of Tennessee Health Science Center Firefighters' Burn Center at Regional One, Memphis, TN; University of Tennessee Health Science Center, Memphis, TN; Firefighters Burn Center, Regional One Health, Memphis, TN; Regional One Health, Memphis, TN; University of Tennessee Health Science Center Firefighters' Burn Center at Regional One, Memphis, TN; University of Tennessee Health Science Center, Memphis, TN; Firefighters Burn Center, Regional One Health, Memphis, TN; Regional One Health, Memphis, TN; University of Tennessee Health Science Center Firefighters' Burn Center at Regional One, Memphis, TN; University of Tennessee Health Science Center, Memphis, TN; Firefighters Burn Center, Regional One Health, Memphis, TN; Regional One Health, Memphis, TN; University of Tennessee Health Science Center Firefighters' Burn Center at Regional One, Memphis, TN; University of Tennessee Health Science Center, Memphis, TN; Firefighters Burn Center, Regional One Health, Memphis, TN

## Abstract

**Introduction:**

After acute burn injury, injured tissue and adjacent non-burned tissue, upregulate response to painful and non-painful stimulus (hyperalgesia and allodynia, respectively). Currently, high-dose fentanyl, oxycodone, hydromorphone, and morphine are used at profound doses to mitigate pain. Oliceridine (OLI), a biased, selective opioid agonist, has shown a 3-fold preferential activation of the G-protein (i.e., analgesia) over β-arrestin pathway. There is no literature of use in patients with burn injuries. We hypothesized the use of OLI would provide adequate and safe analgesia after acute burn injury.

**Methods:**

This single-center, prospective, case-controlled trial was dual IRB approved and included patients with burn injuries admitted between April and September 2023. Informed consent was obtained from the 10 patients receiving OLI. OLI was the only allowed analgesic except for acetaminophen. Dosing and assessments were followed per study protocol and safety and efficacy assessed per study team daily at a minimum. The historical control (CTL) group (i.e., fentanyl, oxycodone, hydromorphone, morphine, etc) was matched 2:1 with the prospective OLI group. The CTL were screened in reverse chronology to find comparable groups according to age, % total body surface area burned (TBSA), history of opioid and illicit drug use, and number of operations. SAS 9.4 was used for descriptive statistics and modelling via UNIVARIATE and GLM procedures.

**Results:**

Two CTL patients were excluded in the final analysis due to missing assessments. The OLI (n = 10) and CTL (n = 18) groups were comparable in age (52.3 ± 12.3 vs 63.1 ± 17.3 years, p = 0.228), TBSA [8 (3.3, 11.2) vs 6 (2.6, 9.3) percent], operations (0.5 per patient for both), and burn mechanism. No patient had a known history of opioid, cocaine, or methamphetamine use. The baseline Numeric Pain Rating Scale (NRS) for OLI and CTL were similar [9 (7.8, 10) vs 9.5 (8.8, 10); p = 0.360); however, the first day’s mean pain score was higher for CTL (4.8 vs. 5.9, p = 0.035). Over the 7-day period, mean daily pain scores significantly declined in patients receiving OLI (-2.65, p = 0.010), but not for CTL (+0.69, p = 0.496). Whereas, opioid usage [i.e., oral morphine milligram equivalents (MME)] was not significantly different across groups (p = 0.689) or days (p = 0.684). Total MME averaged per patient day were 65.2 and 69.6 mg/day in the OLI and control groups, respectively (P = 0.688). There were no unexpected adverse events related to OLI.

**Conclusions:**

Although this sample of 10 patients limits inferences outside of acute burn injuries less than 20% TBSA, OLI demonstrated significant pain relief, which was maintained over the 7 days study period. Importantly, the control group demonstrated initial relief, but was not maintained, despite similar MME.

**Applicability of Research to Practice:**

Oliceridine offers an effective solution for analgesia in patients with acute burn injury.

